# *RET *gene mutations spectrum in patients with medullary thyroid carcinoma (MTC) from Great Poland region

**DOI:** 10.1186/1897-4287-13-S2-A12

**Published:** 2015-11-26

**Authors:** Marta Kaczmarek-Ryś, Katarzyna Ziemnicka, Bartłomiej Budny, Małgorzata Szkudlarek, Szymon Hryhorowicz, Marzena Skrzypczak-Zielińska, Andrzej Pławski

**Affiliations:** 1 Institute of Human Genetics, Polish Academy of Sciences, Poznan, Poland; 2 The Department of Endocrinology, Metabolism and Internal Diseases, University of Medical Sciences, Poznan, Poland; 3 The NanoBioMedical Center, Adam Mickiewicz University, Poznan, Poland

## 

Medullary thyroid carcinoma (MTC) is an epithelial tumor of the thyroid gland, derived from thyroid parafollicular C cells and represents approximately 4% of thyroid carcinomas. In 50 % of patients, MTC is diagnosed at an advanced stage. Due to a lack of TSH receptors on C-cells surface MTC does not respond to suppression therapy with TSH and radio-iodine treatment, and thereby it conferring worse outcomes overall. 5-year survival rates range from 75% for patients with stage III disease to 40% for patients with stage IV disease.

MTC is associated with one of three clinically different, inherited endocrine syndromes caused by germline mutations in the *RET *gene. It encodes a transmembrane receptor tyrosine kinase which is expressed in cells derived from the neural crest.

Activating mutations in the *RET *gene are detected in the majority (95%) patients with an inherited medullary thyroid carcinoma and approximately 30-50% of cases without family history.

Familial medullary thyroid carcinomas (FMTCs) are associated with mutations in codons 609, 611, 618, and 620 (exon 10), as well as codon 768 (exon 13) and codon 804 (exon 14). In FMTC patients, MTC is often the only clinical symptom. Multiple endocrine neoplasia (MEN) 2A is associated in the most cases, with *RET *gene mutations in codons 609, 611, 618, and 620, as well as in codon 634. Patients with MEN2A typically have MTC, pheochromocytoma, and primary hyperparathyroidism (PHPT). Patients diagnosed with MEN 2B present the most aggressive type of MTC, pheochromocytoma but not PHPT, musculoskeletal abnormalities and other developmental defects. This syndrome is associated mostly with mutation in codon 918 in exon 16.

Here we report the *RET *gene mutations spectrum in a group from Great Poland (Wielkopolska) region. Molecular analysis was performed for 304 individuals: patients with diagnosed MTC or apparent MEN 2A syndrome and first and second degree relatives. Studied group included also one family with MEN 2B syndrome. All patients were diagnosed in the Department of Endocrinology, Metabolism, and Internal Diseases of the Poznan University of Medical Sciences. We screened *RET *gene exons: 10, 11, 13-16 using pyrosequencing for specific codons and HRMA technique followed by Sanger sequencing.

Identification of *RET *gene mutations was possible for 50 patients (32%). This group included 12 family cases and in 6 subjected individuals diagnosis were possible before cancer occurring. In a group of 106 (68%) MTC patients we did not detect mutations in analyzed *RET *gene regions.

The most frequent was missense substitutions occurring in codon 634 (72%): C634R (58%), C634G (10%) and C634Y (4%). Surprisingly, in our group of patients we did not detect mutations in exon 10, as they are frequent in other populations. However, we observed 3 different mutations in exon 13 (18%) and in exon 14 (6%). In four unrelated patients with negative family history we detected missense change Y791F, which is associated with late onset MTC, but interestingly one of patients was homozygous and presented more aggressive course of the disease (characterized with multifocality and onset in the moment of diagnosis at 47 years of age). In MEN 2B family, M918A mutation was detected.

Due to the fact, that we observe a different mutational spectrum in our patients, we consider more extensive analysis of the *RET *gene as reasonable.

